# Expression of estrogen receptor, progesterone receptor, human epidermal growth factor receptor 2, and Ki-67 in ductal carcinoma in situ (DCIS) and DCIS with microinvasion

**DOI:** 10.1097/MD.0000000000013055

**Published:** 2018-11-02

**Authors:** Zhi-Bin Wan, Hong-Yi Gao, Lian Wei, An-Qin Zhang, Jiang-Yu Zhang, Yi Wang, Dong-Dong Wang, Yan Zhang

**Affiliations:** a Department of Pathology, Guangdong Women and Children Hospital of Guangzhou Medical University; b Department of Pathology, Guangdong Women and Children Hospital; c School of Public Health, Guangzhou Medical University; d Breast Disease Center, Guangdong Women and Children Hospital, Guangzhou, Guangdong, China.

**Keywords:** breast cancer, ductal carcinoma in situ with microinvasion, ductal carcinoma in situ, molecular subtype

## Abstract

Ductal carcinoma in situ (DCIS) represents a heterogeneous disease in its histologic appearance and biological potential. Some women treated for DCIS subsequently develop invasive breast cancer. DCIS with microinvasion is considered as the interim stage in the progression from DCIS to invasive breast cancer. Analysis of the differences between DCIS and DCIS with microinvasion may aid in understanding the characteristic of DCIS with microinvasion and identifying biological factors determining progression of DCIS to invasive disease.

Retrospective analysis of 219 cases between 2012 and 2018 was performed in our institution. The pathological results and axillary lymph nodes status were collected. Analysis of the expression of estrogen receptor (ER), progesterone receptor (PR), human epidermal growth factor receptor 2 (HER-2), and Ki-67 in pure DCIS (164 cases), and DCIS with microinvasion (55 cases) using immunohistochemistry.

DCIS with microinvasion had a higher nuclear grade (*P* < .001) and was more likely to have sentinel lymph node biopsy (SLNB) positivity (*P* = .039) than DCIS. Expression of ER, PR were significantly higher in DCIS compared with DCIS with microinvasion (*P* < .001, *P* < .001). While the expression of HER-2 in DCIS with microinvasion (56.4%) was significantly higher than in DCIS (36.6%, *P* = .01). Furthermore, DCIS with microinvasion was significantly more likely to have aggressive subtype (Triple-negative and HER2-enriched tumors, *P* = .005).

Our results indicated that DCIS with microinvasion was different from pure DCIS in clinicopathologic characteristics and molecular alterations. It displayed a more aggressive biological nature than pure DCIS. It may be a distinct entity.

## Abstract 2

Supplemental Digital Content is available in the text

## 1 Introduction

Ductal carcinoma in situ (DCIS) is a neoplastic proliferation of epithelial cells growing within the basement membrane-bound structures of the breast and with no evidence of invasion into surrounding stroma.^[1,2]^ Since the introduction of mammography in breast cancer screening, increasing numbers of DCIS are now being identified. It comprises around 20% to 25% of all screening detected breast malignancies.^[3,4]^ DCIS is often described as a noninvasive form of breast cancer or a precursor lesion. However, it represents a heterogeneous disease in its histologic appearance and biological potential.^[1]^ Some DCIS lesions are believed to rapidly transit to invasive breast cancer (IBC), while others remain unchanged.^[5]^ If no treatment is offered, 14% to 46% of patients with DCIS will progress to invasive cancer within 10 years. Approximately one half of all local recurrences that appear after breast-conserving therapy for DCIS are invasive cancers,^[6,7]^ with potential to spread outside of the breast. Invasive recurrence increased subsequent breast cancer mortality 18.1 times.^[8]^ Radiotherapy for DCIS after a complete local excision of the lesion showed a 50% reduction in the risk of local recurrence, but has no effect on breast cancer metastasis and mortality.^[9,10]^ The major gap in our current understanding of DCIS is that we do not know yet which DCIS lesions will develop into invasive breast cancer and which will not. So it is important to ascertain whether the molecular markers could be identified and used to predict DCIS transition to invasive carcinomas and recurrence accurately.

DCIS with microinvasion (<1 mm)^[11]^ is defined as one or several areas of microscopic foci of tumor cells with the invasion of adjacent tissues on the background of DCIS. It included the dominant lesion, which is in-situ carcinoma and one or more foci of infiltration. It is considered as the interim stage in the progression from DCIS to invasive breast cancer.^[12]^ Recent studies revealed that DCIS with microinvasion was potential for invasion and metastasis differentiated from pure DCIS, which also resulted for the different surgical strategy.^[13]^ The aim of the study was to analyze the difference of clinicopathological characteristics and molecular phenotypes in DCIS and DCIS with microinvasion, and furthermore to predict patients most at risk of disease progression, avoiding over- or under-treatment.

## 2 Materials and methods

### 2.1 Patients

A total of 219 patients from the Guangdong Women and Children Hospital between January 2012 and January 2018 were enrolled in this study. Among these cases, 164 cases were diagnosed pure DCIS, 55 cases were confirmed DCIS with microinvasion (<1 mm) by immunohistochemistry. All the subjects were Chinese women patient treated for the first time. None of them had received any treatment before the biopsy procedure. Tissue samples were from the patients undergoing lumpectomy or mastectomy. Histopathological classification was performed on the basis of the current diagnostic criteria of the World Health Organization classification.^[11]^ Previous written and informed consent were obtained from every patient and the study was approved by the Research Ethics Committee of Guangdong Women and Children Hospital.

### 2.2 Immunohistochemistry staining and fluorescence in situ hybridization (FISH)

All tissue samples had been routinely fixed in 10% neutral buffered-formalin and embedded in paraffin within 24 to 48 hours. Immunohistochemical staining were performed separately with an automatic staining device (BenchMark XT, Ventana Medical Systems, Tucson, AZ), using optimally formulated rabbit monoclonal primary antibodies (Ventana Medical Systems) to estrogen receptor (ER) (SP1), progesterone receptor (PR) (1E2), human epidermal growth factor receptor 2 (HER-2) (4B5), and Ki-67 (MIB-1). Dual-probe FISH was carried out for those cases with score 2+ by immunohistochemistry (IHC). Detection procedures followed the manufacturer's instructions for FISH kit for the detection of HER-2 amplification (GP Medical Technologies, Beijing, China).

### 2.3 Interpretation of staining

**Figure 1:**
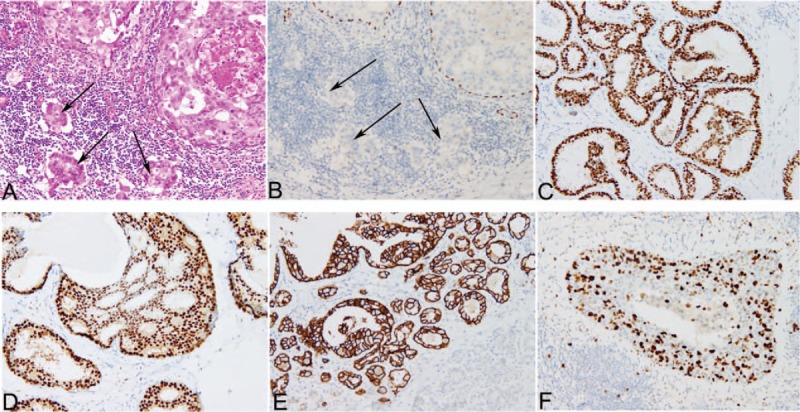
Hematoxylin-eosin and immunohistochemical staining of tissues from the cases. A, Hematoxylin-eosin staining of the case with DCIS with microinvision. Two ducts are filled by ductal carcinoma in situ, while the small clusters of carcinoma cells (<1 mm) invade the stroma (arrows), 200×. B, Immunohistochemical staining for p63 highlights continuous positivity in myoepithelial cells of DCIS, while absence of myoepithelial cells around the tumor cell clusters confirms microinvasion (arrows), 200×. C–F, Immunohistochemical staining of ER (C), PR (D), HER-2 (E) and Ki-67 (F), 200×. DCIS = ductal carcinoma in situ, ER = estrogen receptor, HER-2 = human epidermal growth factor receptor 2, PR = progesterone receptor.

The immunohistochemistry results were evaluated independently by 2 pathologists. ER and PR assays were considered positive if there are at least 1% positive tumor nuclei in the sample on testing in the presence of expected reactivity of internal (normal epithelial elements) and external controls. ER or PR were considered negative if <1% of tumor cell nuclei were immunoreactive in the presence of evidence that the sample can express ER or PR (positive intrinsic controls seen).^[14]^ Interpretation of HER-2 staining was according to the literature.^[15]^ HER-2 positivity was considered as score 3+ by IHC or FISH positive, whereas cases with score 0 to 1+ or 2+ without FISH positive were regarded as negative. Proliferation was considered high if IHC staining for Ki-67 was seen in >20% of tumor nuclei (Fig. 1C–F).^[16]^

### 2.4 Statistical analyses

Data were analyzed using SPSS16.0 statistical software (SPSS Inc, Chicago, IL). The Chi square test or Fisher exact test (the expected value in any cell was <5) were used as appropriate. All tests carried out were 2 sided. *P* < .05 was considered statistically significant.

## 3 Results

### 3.1 Patients and tumor characteristics

The clinicopathological characteristics of the DCIS and DCIS with microinvasion patients were showed in Table 1. A total of 219 cases were included in the study (supplementary table). These were all newly diagnosed either through the screening program or as symptomatic cases. All the patients were women, and the median age was 46 years (range 21–75 years). Among these cases, 164 cases were diagnosed pure DCIS, 55 cases were confirmed DCIS with microinvasion using immunostains for myoepithelial markers (Fig. 1A and B), 3 cases were with paget disease of the nipple. Tumor size ranged from 0.2 to 14.0 cm. The sentinel lymph node biopsy (SLNB) was performed in 72 (32.9%). Metastasis was present in 5 patients, and 4 of them were with micrometastasis. Furthermore, 5 patients underwent axillary lymph node dissection (ALND) and no metastasis was found.

**Table 1:**
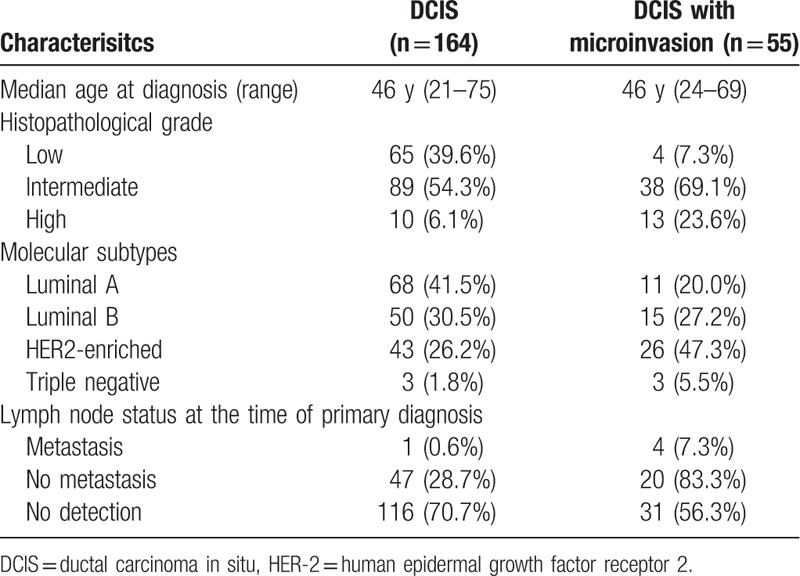
Clinicopathologic characteristics of the full cohort (n = 219).

### 3.2 Comparison of clinical parameters and biomarkers between DCIS and DCIS with microinvasion

We compared clinical parameters of the DCIS patients with those showing DCIS with microinvasion. As shown in Table 2, the proportion of “intermediate to high nuclear grade” tumors was larger in DCIS with microinvasion (92.7%) compared with that in DCIS (60.4%, *P* < .001). In 164 cases with DCIS, metastasis was present in one of 48 (2.1%) patients undergoing lymph node biopsy. While in DCIS with microinvasion, it was16.7% (4/24), and was significantly higher than in DCIS (*P* = .039). No differences were observed between DCIS and DCIS with microinvasion for age distribution (*P* = .669).

**Table 2:**
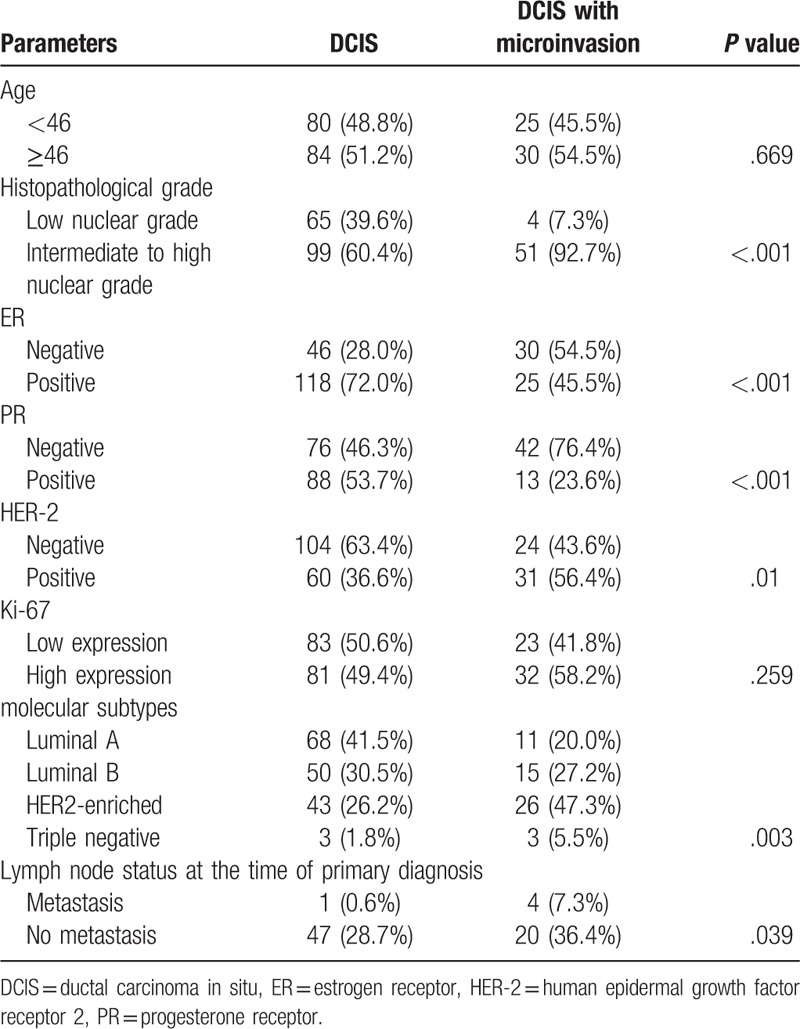
Expression of ER, PR, HER-2, and Ki-67 in DCIS and DCIS with microinvasion.

ER showed an expression in 72.0% (118/164) in DCIS. It was significantly higher than that in DCIS with microinvasion (45.5%, *P* < .001). Similarly, PR was positive in 53.7% (88/164) versus 23.6% (13/55) of DCIS and DCIS with microinvasion, with significant statistical differences (*P* < .001). HER-2 amplification was demonstrated in 36.6% of DCIS. Interestingly, the HER-2 expression in DCIS with microinvasion (56.4%) was significantly high than in DCIS. No differences were observed between DCIS and DCIS with microinvasion for Ki-67 expression (*P* = .259). All the case could be classified into molecular subtypes using 1% and 20% as cut-offs for steroid receptor status and proliferation, respectively. The Luminal A and Luminal B subtypes were more often present in DCIS, while patients of DCIS with microinvasion more likely to have HER-2+ and Triple Negative type tumors or less likely Luminal A and Luminal B type tumors. These differences between 2 groups were statistical significance (*P* = .003).

## 4 Discussion

DCIS is often described as a noninvasive form or preinvasive lesion of breast cancer. Nevertheless, it is known that some women treated for DCIS subsequently develop invasive breast cancer, which is associated with a poorer prognosis. The step-wise transformation events that drive its progression are unknown. Microinvasion is considered to be the interim stage in the progression from DCIS to invasive breast cancer. It is a rare subset of breast carcinoma comprising 0.7% to 2.4% of all patients with breast cancer^[17]^ and is described as the smallest morphologically identifiable stage of invasion. Its presence and distinction from in situ carcinoma may have therapeutic implications, and clinical staging also requires the recognition of this phenomenon. This study attempted to study the differences of clinicopathological features and molecular biomarkers among DCIS and DCIS with microinvasion, in order to describe the process of DCIS to IBC from the clinical aspects and molecular mechanism, furthermore to make a appropriate treatment. In patients with microinvasion, the most commonly seen component in the background was high-grade DCIS, and it is very unusual in low-grade disease.^[18]^ Previous study showed patients with DCIS with microinvasion were more likely with comedo-type necrosis, or with high nuclear grade than patients with DCIS.^[19]^ In this study, we found that there was significant difference between patients with DCIS and DCIS with microinvasion in nuclear grade. The high nuclear grade presented more often in DCIS with microinvasion. Kerlikowske et al^[20]^ reported that nuclear grade was an independent predictor of local recurrence in DCIS. These suggested that DCIS with microinvasion may tend to recurrence and have poor prognosis.

DCIS usually is considered noninvasive with theoretically no potential for lymph node or distant metastases by definition. Occasionally, an increased risk of invasion and metastasis exists. The SLNB is a minimally invasive procedure. It can be used for identification of patients at higher risk for lymph node metastases and decrease unnecessary axillary surgery in low-risk patients. Reports from large amount of institutes, the rate of mastectomy and ALND decreased significantly, whereas an increased rate of breast conservative surgery (BCS) and SLNB in patients with DCIS or DCIS with microinvasion was showed dramatically about a decade ago. The decision to perform an SLNB should be based on the underlying risk of invasion. The risk increases with the presence of a palpable mass, intermediate or high grade DCIS, younger age, and extensive microcalcifications. The risk of a positive sentinel lymph node (SLN) with pure DCIS is small (0.39%–13.7%) and most of the metastases found are micrometastases or isolated tumor cells, detected by immunohistochemistry.^[21,22]^ In 3 large series, the authors concluded that a microinvasive lesion shown on biopsy or an invasive component shown by surgery significantly increased the risk of positive SLN. Klauber-De More et al^[23]^ reported 12% SLN positivity in DCIS. But when patients with microinvasive focus and patients with stromal and vascular invasion were excluded, the incidence decreased to 6.5%. Ozkan-Gurdal et al^[19]^ reported that 3.3% was found to have isolated tumor cells in SLNB of patients with pure DCIS, whereas 7.1% of patients having DCIS with microinvasion had micrometasis in SLNB. In concordance with such previous studies, we also found a low rate of SLN involvement (2.1%) in patients with pure DCIS, and a relatively higher incidence of lymph node involvement (16.7%) in patients with DCIS with microinvasion. Four of all cases (4/5) with SLN positive were micrometastasis. The results indicated that DCIS with microinvasion were favor to appear SLN positive. However, more advanced research and large series are needed to confirm it.

As we know, ER, PR, HER-2, and Ki-67 are important in invasive breast cancer. They are not only as prognostic markers, but also as predictors of response to therapy. In DCIS, these biomarkers may also be anticipated to reflect disease biological behavior. It was reported that expression of ER is strongly associated with low grade DCIS, while HER-2 overexpression is strongly associated with high grade disease. Ozkan-Gurdal et al^[19]^ reported that DCIS with hormone receptor negativity was more likely to have a microinvasive component in multivariate analysis. A recent study found overexpression of HER-2 in DCIS to be the only significant predictor of invasive disease in a multivariate analysis, and suggested that HER-2 may be important in promoting invasion. Roses et al^[24]^ demonstrated that although high nuclear grade, large lesion size, and HER-2 overexpression were all associated with the presence of invasive disease on univariate analysis. HER-2 was the only significant predictor for the presence of invasive disease after multivariate adjustment. In this study, we demonstrated that expression of ER, PR were significant higher in DCIS compared with DCIS with microinvasion, while HER-2 was overexpression in DCIS with microinvasion. The results are consistent with previous studies.^[25]^ This indicating that subsets of DCIS and DCIS with microinvasion may be different molecularly. Moreover, ER, PR, and HER-2 expression may reflect an important pathway through which DCIS lesions may progress toward invasion.

Ki-67 is a nuclear protein used as a proliferation marker and a strong prognostic indicator for poorer outcome in early, node negative invasive breast cancer. Accordingly, our findings showed that Ki-67 had a relatively high expression in DCIS with microinvasion compared with DCIS. However, the difference between 2 patients groups did not reach statistical significance.

The expression of ER, PR, HER-2, and Ki-67 have been used to discriminate different molecular subtypes (Luminal A, Luminal B, HER-2-enriched, and Triple-negative).^[26]^ Similar molecular phenotypes seen in invasive cancer presented in primary DCIS using immunohistochemistry surrogate markers.^[27,28]^ Patients with DCIS with microinvasion in our study were significantly more likely to have HER-2-enriched and Triple-negative tumors or less likely Luminal A and Luminal B type tumors than patients with DCIS. This is consistent with previous studies.^[29]^ Triple-negative and HER2-positive tumors are both known to be aggressive phenotypes. This indicated that DCIS with microinvasion exhibited more aggressive biological behavior than pure DCIS.

In conclusion, our results indicated that DCIS with microinvasion was different from pure DCIS in clinicopathologic characteristics and molecular alterations. It had a higher nuclear grade and was more likely to have SLNB positivity. It also displayed more aggressive molecular subtype. It may be a distinct entity. Therapy of DCIS with microinvasion needs to be further optimized. Furthermore, further research is needed to understand DCIS with microinvasion and identify biological factors determining progression of DCIS to invasive disease.

## Author contributions

**Conceptualization:** Yan Zhang.

**Data curation:** Zhi-Bin Wan, Lian Wei, Jiang-Yu Zhang, Yi Wang.

**Formal analysis:** Hong-Yi Gao.

**Funding acquisition:** Yan Zhang.

**Investigation:** Hong-Yi Gao, Lian Wei, An-Qin Zhang, Jiang-Yu Zhang, Yi Wang, Dong-Dong Wang.

**Writing – original draft:** Zhi-Bin Wan, Yan Zhang.

**Writing – review & editing:** Yan Zhang.

## Supplementary Material

Supplemental Digital Content
